# Intestinal microbiota dysbiosis contributes to the liver damage in subchronic arsenic-exposed mice: Intestinal microbiota imbalance of arsenic-induced hepatotoxicity in mice

**DOI:** 10.3724/abbs.2024131

**Published:** 2024-10-08

**Authors:** Ling Dong, Peng Luo, Aihua Zhang

**Affiliations:** The Key Laboratory of Environmental Pollution Monitoring and Disease Control Ministry of Education Department of Toxicology School of Public Health Collaborative Innovation Center for Prevention and Control of Endemic and Ethnic Regional Diseases Co-Constructed by the Province and Ministry Guizhou Medical University Guiyang 561113 China

**Keywords:** arsenic, intestinal microbiota, fecal microbiota transplantation, intestinal barrier, liver damage

## Abstract

There is an extensive amount of evidence that links changes in the intestinal microbiota structure to the progression and pathophysiology of many liver diseases. However, comprehensive analysis of gut flora dysbiosis in arsenic-induced hepatotoxicity is lacking. Herein, C57BL/6 mice are exposed to arsenic (1, 2, or 4 mg/kg) for 12 weeks, after which fecal microbiota transplantation (FMT) study is conducted to confirm the roles of the intestinal microbiome in pathology. Treatment with arsenic results in pathological and histological changes in the liver, such as inflammatory cell infiltration and decreased levels of TP and CHE but increased levels of ALP, GGT, TBA, AST, and ALT. Arsenic causes an increase in the relative abundance of *Escherichia-Shigella*, *Klebsiella* and *Blautia*, but a decrease in the relative abundance of *Muribaculum* and *Lactobacillus*. In arsenic-exposed mice, protein expressions of Occludin, ZO-1, and MUC2 are significantly decreased, but the level of FITC in serum is increased, and FITC fluorescence is extensively dispersed in the intestinal tract. Importantly, FMT experiments show that mice gavaged with stool from arsenic-treated mice exhibit severe inflammatory cell infiltration in liver tissues. Arsenic-manipulated gut microbiota transplantation markedly facilitates gut flora dysbiosis in the recipient mice, including an up-regulation in *Escherichia-Shigella* and *Bacteroides*, and a down-regulation in *Lactobacillus* and *Desulfovibrio*. In parallel with the intestinal microbiota wreck, protein expressions of Occludin, ZO-1, and MUC2 are decreased. Our findings suggest that subchronic exposure to arsenic can affect the homeostasis of the intestinal microbiota, induce intestinal barrier dysfunction, increase intestinal permeability, and cause damage to liver tissues in mice.

## Introduction

Arsenic is a toxic metalloid that widely occurs naturally in the environment, and a study estimated that approximately 220 million people are likely to be exposed to arsenic (high level) in groundwater [1]. Long-term exposure to environmental arsenic can result in multiorgan system damage. The liver is the primary organ affected by arsenic toxicity [2]. Substantial evidence revealed that arsenic exposure can induce hepatotoxicity, including liver injury, inflammation [ 3, 4], hepatic fibrosis [5], and liver tumors [6], thus accelerating the progression of liver damage. Studies related to toxic environmental pollutants, such as lead, 4-tert-butylphenol, chlorpyrifos, and arsenic, suggested that various pathogenic factors participate in liver damage progression, including oxidative damage [ 7, 8], inflammation [9], apoptosis, necroptosis [10], autophagy, mitophagy [11], imbalanced immunity [ 4, 12], imbalanced elements [ 13, 14], and disrupted gut flora [15].

The gut-liver axis is a bidirectional information interaction system between the liver and the gastrointestinal tract [ 16, 17], in which the balance of intestinal microbiota plays a key role. Perturbations of intestinal flora play a vital role in the progression of many liver diseases [18]. Gut microbiota comprises approximately 100 trillion microbes, and these microorganisms are related to the regulation of host immunity, metabolism, and diseases [19]. The gut microbiome composition is vital for preserving the balance of the gut-liver axis, and as part of the crosstalk, liver moulds the gut flora communities [20]. The intestinal microbiota has attracted widespread attention because it plays an extremely important role in the pathogenesis of liver diseases [ 19 , 21]. Importantly, the gut flora has been shown to contribute to the pathogenesis of numerous diseases, such as depressive disorder [22], Crohn’s disease [23], obesity [24], hypertension [25], diabetes [26], and liver diseases [27], through fecal microbiota transfer and gut flora reshaping experiments. Exploring the changes in the components of the gut flora will provide novel therapeutic targets and strategies for arsenic-induced liver damage. Previous studies have shown that the intestinal flora of mice exposed to arsenic differs from that of normal mice [ 28, 29]. However, whether transplantation of mice exposed to arsenic in the fecal microbiota can reproduce hepatotoxicity and underlying mechanism have not been explored.

In this study, we conducted comprehensive 16S rDNA sequencing analysis in mice exposed to arsenic and then performed fecal microbiota transplantation (FMT) from mice exposed to arsenic to recipient mice after antibiotic treatment. The data of the present research may be helpful for understanding the potential mechanisms of arsenic-induced hepatotoxicity and laying a foundation for future research.

## Materials and Methods

### Chemicals

Sodium arsenite (NaAsO _2_, ≥ 90.0%) was purchased from Sigma-Aldrich (St Louis, USA). Neomycin sulfate (N8090), ampicillin (A6920), vancomycin hydrochloride (V8050), metronidazole (M8060), sterile saline (IN9000), brain heart infusion agar (BHIA, LA0680), Maconkey agar (MAC, M8560), hematoxylin and eosin kits (H&E, G1121), and alcianblue-periodic acid schiff kits (AB-PAS, G1285) were purchased from Solarbio (Beijing, China). Total protein (TP, A045-1-1), albumin (ALB, A028-2-1), choline esterase (CHE, A023-2-1), gamma-glutamyl transferase (GGT, C017-2-1), alkaline phosphatase (ALP, A059-2-2), total bile acid (TBA, E003-2-1), alanine aminotransferase (ALT, C009-2-1), and aspartate transaminase (AST, C010-2-1) kits were supplied by Jiancheng Bioengineering Institute (Nanjing, China). Antibodies against Occludin (abs136990), ZO-1 (abs131224), MUC2 (abs154698), and β-actin (abs132001) were purchased from Absin Science & Technology (Shanghai, China). HRP-conjugated goat anti-rabbit IgG (H+L) antibody (RS0002) was supplied by Immunoway Biotechnology (Beijing, China). All chemicals used were of analytical grade.

### Arsenic exposure model in mice

Male ( *n*=16) and female ( *n*=16) C57BL/6 mice of specific pathogen-free (SPF) grade, aged 8 weeks, provided by Huafukang Biotechnology (Beijing, China) with a National Animal Use Licence number of SCXK(Jing)-2019-0008, were used in this study and randomly assigned to 4 groups (eight mice per group) using a random number table method and adapted for 1 week before the assay. First, thirty-two mice were divided into 4 groups by daily oral gavage as follows: (i) the normal control group (NC, 0 mg/kg NaAsO _2_), (ii) the low NaAsO _2_ group (L-As, 1 mg/kg NaAsO _2_), (iii) the medium NaAsO _2_ group (M-As, 2 mg/kg NaAsO _2_), and (iv) the high NaAsO _2_ group (H-As, 4 mg/kg NaAsO _2_); the arsenic intervention lasted for 12 weeks. The doses of NaAsO _2_ used were selected on the basis that the half lethal dose (LD _50_) of NaAsO _2_ in mice is 16.5 mg/kg [30]. According to the principle of subchronic toxicity testing, the L-As, M-As, and H-As groups were orally exposed to 1 mg/kg (1/16 LD _50_), 2 mg/kg (1/8 LD _50_), or 4 mg/kg (1/4 LD _50_) NaAsO _2_, respectively. All mice were fed with a normal diet (Huanyu Biotechnology, Beijing, China) throughout the entire experimental period. After gavage for 12 weeks, the stool of mice was gathered for 16S rDNA sequencing analysis. Subsequently, the fresh stool was used for the FMT experiment.

### FMT experiment in a pseudogerm-free mouse model

Thirty-two C57BL/6 mice (half male and half female) of SPF grade, aged 8 weeks, were used in the present study and randomly assigned to 4 groups (eight mice per group) using a random number table method and adapted for 1 week before any treatment. For microbiota transplantation, stool was collected from donors (mice exposed to arsenic), levitated with sterile saline, and centrifuged as previously described [25]. First, thirty-two mice that were administered filter-sterilized water supplemented with antibiotics, including 0.5 g/L vancomycin, 1 g/L ampicillin, 1 g/L neomycin sulfate, and 1 g/L metronidazole, for 2 weeks to remove the gut microbiota [26] were randomly assigned to the (i) FMT normal control (FMT-NC) group, (ii) FMT low NaAsO _2_ (FMT-LA) group, (iii) FMT medium NaAsO _2_ (FMT-MA) group, and (iv) FMT high NaAsO _2_ (FMT-HA) group. For FMT, feces from the NC, L-As, M-As, and H-As groups were harvested daily and transferred to the FMT-NC, FMT-LA, FMT-MA, and FMT-HA groups, respectively. Finally, 200 μL of the supernatant microbiota was gavaged to pseudogerm-free mice for 12 weeks. Body weights were measured weekly. The experimental animals were raised in a controlled environment (12 h light-dark cycle, relative humidity 60%±5%, temperature 22°C±2°C) with *ad libitum* access to water and a normal chow diet (Huanyu Biotechnology). All animal procedures were approved by the Animal Experimental Ethical Committee of Guizhou Medical University with an approval number 1403059.

### Bacterial culture

After treatment with antibiotics for 2 weeks, the stool of pseudogerm-free mice was isolated, collected, and homogenized in 900 μL of aseptic PBS and 0.1 g of feces. A drop of the suspension was surrounded on BHIA agar and cultured at 37°C for 24 h. The next day, the reproduced bacterial colonies were observed.

### Histopathological evaluation of intestinal and liver tissues

For histopathological observation, the liver and gut tissues were fixed in 10% neutral formaldehyde for 48 h, dehydrated, embedded in paraffin blocks to make conventional paraffin sections, and sectioned at 4 μm with a microtome (RM 2135; Leica, Wetzlar, Germany). After H&E staining, the specimens were examined under a Nikon microscope (Olympus, Tokyo, Japan). Pathological examination of the colonic mucosa was performed by AB-PAS staining.

### Determination of liver function

The serum levels of TP, ALB, CHE, GGT, ALP, TBA, ALT, and AST were measured using biochemical kits according to the instructions of the corresponding kits. Briefly, serum was added to the 96-well plate supplied with the kit, then the reaction solution was added, the liver parameter levels were detected with a microplate reader (SuPerMax 3100; Flash Spectrum Biochem Technologies, Shanghai, China) after incubation.

### Evaluation of the intestinal microbiota by 16S rDNA sequencing

In the present study, the process used to perform intestinal flora profiling on mouse stool samples included the following: (i) collection of stool samples, (ii) isolation of DNA from bacteria in stool samples, (iii) selection of the 16S rDNA sequencing target, and (iv) analysis of the 16S rDNA sequence data. Sequencing was conducted by LC-Bio (HangZhou, China).

### Western blot analysis

For intestinal barrier-related protein determination, a western blot analysis was performed according to the methods of a previous study [4]. First, proteins were extracted from gut tissues, after which the proteins were separated via 10% sodium dodecyl sulfate-polyacrylamide gel electrophoresis (SDS-PAGE) and transferred onto polyvinylidene difluoride (PVDF) membranes (Millipore, Billerica, USA). After being blocked, the membranes were incubated with primary antibodies (Absin Science & Technology), including rabbit anti-ZO1 polyclonal antibody (1:2000), rabbit anti-Occludin polyclonal antibody (1:2000), rabbit anti-MUC2 monoclonal antibody (1:2000), and rabbit anti-β-actin polyclonal antibody (1:10,000), at 4°C overnight. The following day, the membranes were incubated with secondary antibody (1:8000) for 2 h at room temperature. The membranes were visualized via ECL Millipore (E8059) and analyzed using an Image Lab system (Bio-Rad, Hercules, USA). The data are expressed as the protein level normalized to that of β-actin.

### Immunohistochemistry analysis

The details and steps of the immunohistochemical analysis were described in our previous study [4]. Briefly, colon tissues were fixed, dehydrated, embedded in paraffin, and sectioned, and then, the sectioned colon tissues were incubated with primary antibodies, including rabbit anti-ZO1 polyclonal antibody (1:50), rabbit anti-Occludin polyclonal antibody (1:100), and rabbit anti-MUC2 monoclonal antibody (1:100), at 4°C overnight. The next day, the colon tissues were incubated with secondary antibody. A diaminobenzidine (DAB; Zhongshan Biotechnology, Beijing, China) assay kit was used to observe the localization of Occludin, ZO-1, and MUC2. All the slides were observed under a Nikon microscope (Olympus, Tokyo, Japan), and analyzed with Image-Pro Plus version 6.0 software.

### Evaluation of intestinal permeability

Intestinal permeability was assessed using an *in vivo* 4 kDa fluorescein isothiocyanate-dextran (FITC) permeability assay. Mice were administered 600 mg/kg body weight 4 kDa FITC (FD40S; Sigma-Aldrich) in sterile saline by oral gavage. One and half an hour later, a small animal imaging system (IVIS Lumina Series III; PerkinElmer, Shelton, USA) was used to observe the dispersion of FITC in the intestinal tract of the mice, and 4 h later, blood samples were collected from the venae canthus medialis and centrifuged at 900 *g* for 15 min at 4°C to obtain serum samples. The serum was diluted with sterile saline, and then, the FITC levels were examined with the microplate reader at an excitation/emission wavelength of 485 nm/528 nm. Moreover, the livers, mesenteric lymph nodes (MLNs), and spleens of the mice were isolated and homogenized with 900 μL of aseptic PBS and 0.1 g of tissue. A drop of suspension was spread on MAC agar and cultured at 37°C for 24 h. Then, the reproduced bacterial colonies were observed.

### Statistical analysis

Data are expressed as the mean±standard deviation (SD). One-way analysis of variance (ANOVA) and Student’s *t*-test were used to assess the differences between the groups. The statistical analysis was conducted using IBM SPSS version 26.0 and GraphPad Prism version 8.3. The results were considered statistically significant at *P*<0.05.

## Results

### Arsenic exposure induces liver damage in mice

**Figure 1:**
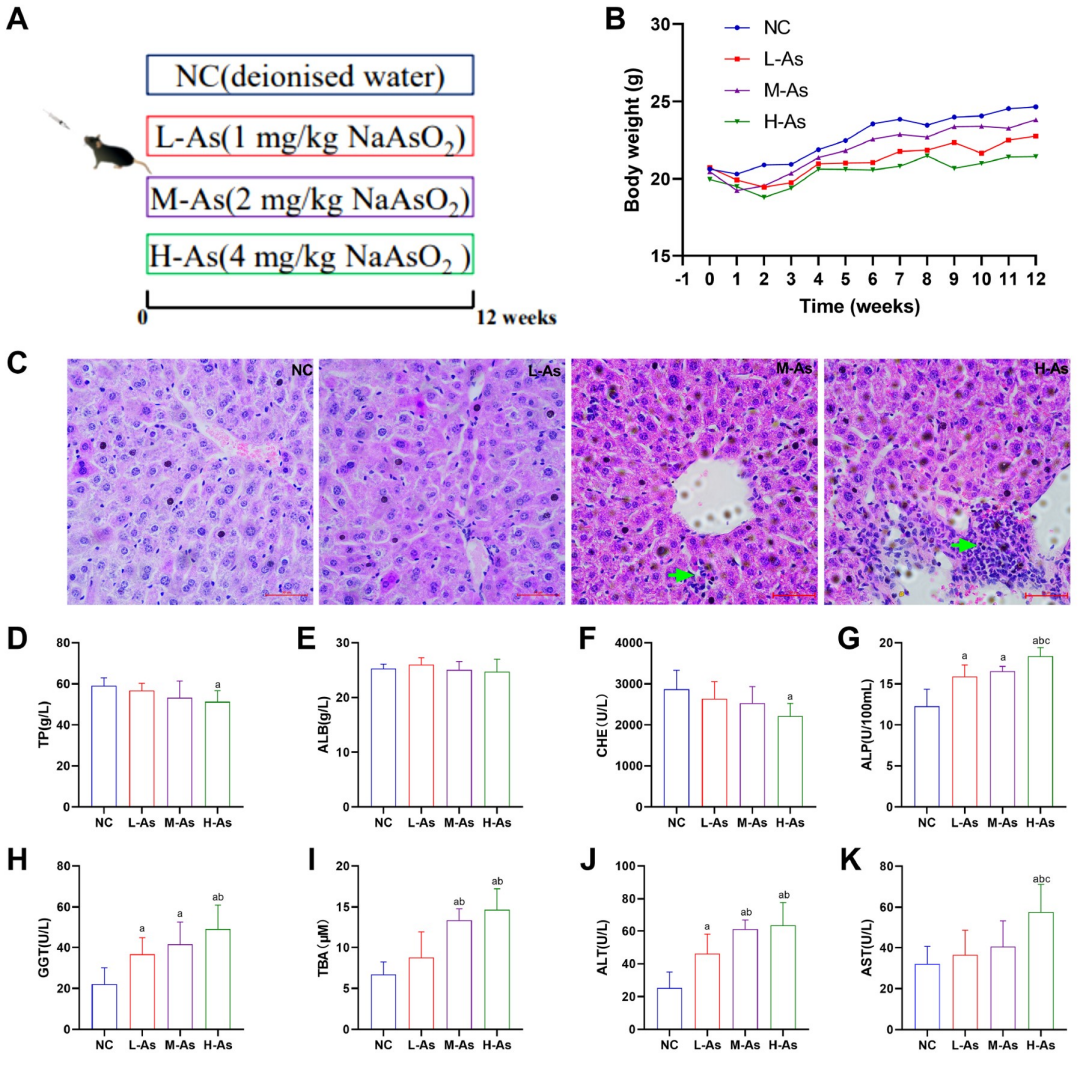
Figure 1 Arsenic causes hepatic histopathological and serum biochemical changes (A) The specific animal groups and administration methods. (B) Body weight changes. (C) Liver pathological changes (magnification 400×; scale bar: 50 μm). The green arrows represent inflammatory cell infiltration. (D) TP content. (E) ALB content. (F) CHE activity. (G) ALP activity. (H) GGT activity. (I) TBA content. (J) ALT activity. (K) AST activity. Data are expressed as the mean±SD; n=4–6 for each group. aP<0.05 compared with the NC group; bP<0.05 compared with the L-As group; cP<0.05 compared with the M-As group.

To validate whether the model of liver damage caused by arsenic was successful established ( Figure 1A), the changes of body weight, histopathology of liver tissues, and biochemical indicators of hepatotoxicity in mice were observed. Figure 1B showed that the differences in body weight between the control and arsenic-treated mice were not statistically significant ( *P*>0.05). As shown in Figure 1C, inflammatory cell infiltration was detected in the livers of the arsenic-exposed mice. As shown in Figure 1D–K, biochemical indicators of hepatotoxicity (ALP, GGT, TBA, ALT, and AST) were upregulated in the arsenic exposure groups ( *P*<0.05). Compared with the L-As group, the levels of ALP, GGT, TBA, ALT, and AST increased in H-As group ( *P*<0.05). However, compared with those in the NC group, the contents of TP and CHE in the H-As group were decreased ( *P*<0.05). No significant differences were found in the content of the ALB among these groups ( *P* >0.05).

### Arsenic exposure induces intestinal microbiota dysbiosis in mice

**Figure 2:**
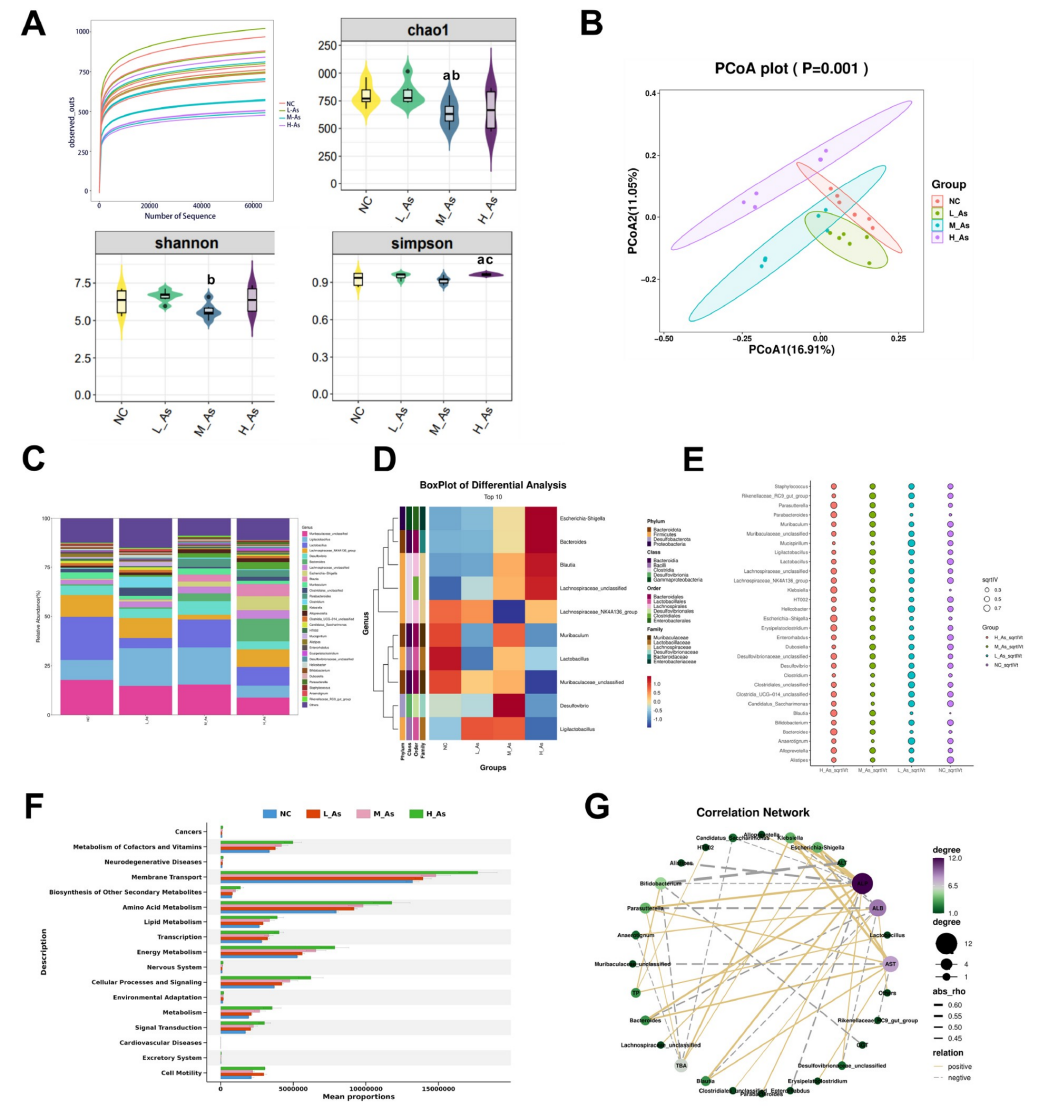
Figure 2 Gut microbiota dysbiosis in mice induced by arsenic (A) Accumulation curves and alpha diversity. (B) Principal coordinate analysis (PCoA) of weighted UniFrac distances. (C) The relative abundance of the intestinal microbiome at the genus level. (D) Heatmap of the intestinal microbiome species among the groups. (E) Indicator analysis. (F) PICRUSt software was used to predict the functions of the microbial genes. (G) The correlation between liver function indicators and microbiota composition in arsenic-induced liver damage mice. aP<0.05 compared with the NC group; b P<0.05 compared with the L-As group; cP<0.05 compared with the M-As group.

To evaluate the effects of arsenic exposure on the balance of intestinal microbiota, the stool samples in mice were evaluated by 16S rDNA sequencing. As shown in Figure 2A, a gentle dilution curve confirmed that the amount of sequencing data was saturated. The Chao1 indices in M-As group were lower than those in NC and L-As group ( *P*<0.05). The changes in Shannon indices between the M-As and L-As group were different ( *P*<0.05). In addition, the Simpson indices in H-As group were higher than those in NC and M-As group ( *P*<0.05), suggesting that arsenic-induced hepatotoxicity influenced the abundance of the gut flora in the mice.

Principal component analysis (PCoA) of the gut flora in the mice from different arsenic supplementation groups demonstrated that distinct separations were observed in the PCoA among the NC, L-As, M-As, and H-As groups, indicating a significant change in the gut microbiota composition induced by arsenic ( Figure 2B).

The relative abundances of the top 30 intestinal microbes were shown in Figure 2C. At the genus level ( Figure 2D) **,** the gut flora species among the groups were not obviously the same, arsenic increased the abundances of *Escherichia-Shigella*, *Bacteroides* , *Blautia*, and *Parasutterella* in the mice. However, compared to those in the NC group, the relative abundances of *Muribaculum*, *Lactobacillus*, *Ligilactobacillus*, and *Muribaculaceae_unclassfied* in the arsenic exposure groups were decreased.

Furthermore, we screened for arsenic exposure-associated biomarkers. Figure 2E showed the relative abundance of altered gut bacterial genera and the indicators. The differences in abundance were observed for *Parabacteroides*, *Klebsiella*, *Escherichia-Shigella*, *Blautia*, and *Bacteroides* in the NC group, indicating that *Parabacteroides*, *Klebsiella*, *Escherichia-Shigella*, *Blautia*, and *Bacteroides* could be biomarkers of arsenic exposure.

On the basis of the gut flora composition obtained by 16S rDNA sequencing, PICRUSt software was “mapped” to the KEGG database to predict the functions of the microbial genes. Arsenic induced changes in membrane transport, amino acid metabolism, lipid metabolism, energy metabolism, and cellular processes and signaling ( Figure 2F). Pearson’s correlation analysis between fecal bacteria and liver function parameters revealed that the contents of arsenic exposure-enriched gut bacteria, such as *Escherichia-Shigella*, *Blautia*, *Klebsiella* , *Parasutterella*, and *Bacteroides*, were significantly positively correlated with the serum levels of ALP and AST but negatively correlated with the level of ALB ( *P*<0.05). Additionally, the abundances of arsenic-reducing gut bacteria, such as *Lactobacillus*, *Bifidobacterium*, and *Anaerotignum* were positively correlated with the serum parameters TP and ALB but negatively correlated with ALP, AST, and TBA ( *P*<0.05) ( Figure 2G).

These data indicated that subchronic exposure to arsenic can affect the homeostasis of the intestinal microbiota, which might be correlated with the liver damage in mice.

### Effects of arsenic exposure on gut histopathology in mice

**Figure 3:**
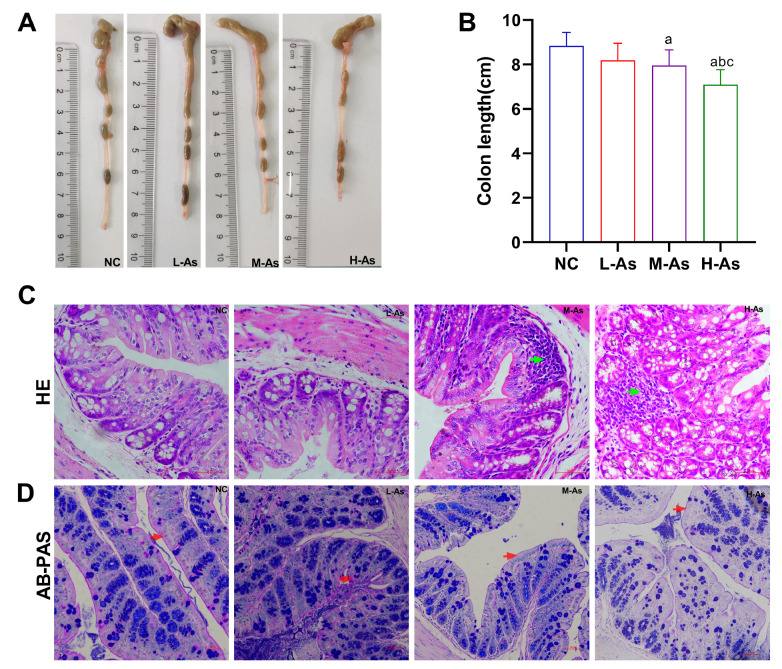
Figure 3 Arsenic causes gut histopathological changes (A) Colon length. (B) Colon length (bar chart). (C) Pathological changes in the colon (magnification 400×; scale bar: 50 μm). The green arrows indicate inflammatory cell infiltration. (D) AB-PAS staining of the colonic mucosa (magnification 200×; scale bar: 50 μm); the red arrows indicate the mucin layer. Data are expressed as the mean±SD; n=4–6 for each group. a P<0.05 compared with the NC group; bP<0.05 compared with the L-As group; cP<0.05 compared with the M-As group.

H&E staining was conducted to examine the intestinal morphological alterations in colon tissue, and mucosal changes were detected using AB-PAS staining. The colon length was shorter in the exposure groups than in the NC group ( *P*<0.05; Figure 3A,B). The morphological changes were presented in Figure 3C. The colon tissues of the NC mice exhibited well-arranged epithelial cells and a normal morphology. However, mice exposed to 2 mg/kg and 4 mg/kg arsenic showed inflammatory cell infiltration. The AB-PAS staining results showed that the intestinal mucosa of the NC mice was covered with a thicker mucin layer ( Figure 3D, red arrows). However, after exposure to arsenic, the mucin layer became thinner. These results showed that arsenic caused gut damage in mice.

### Effects of arsenic on the intestinal barrier and permeability in mice

**Figure 4:**
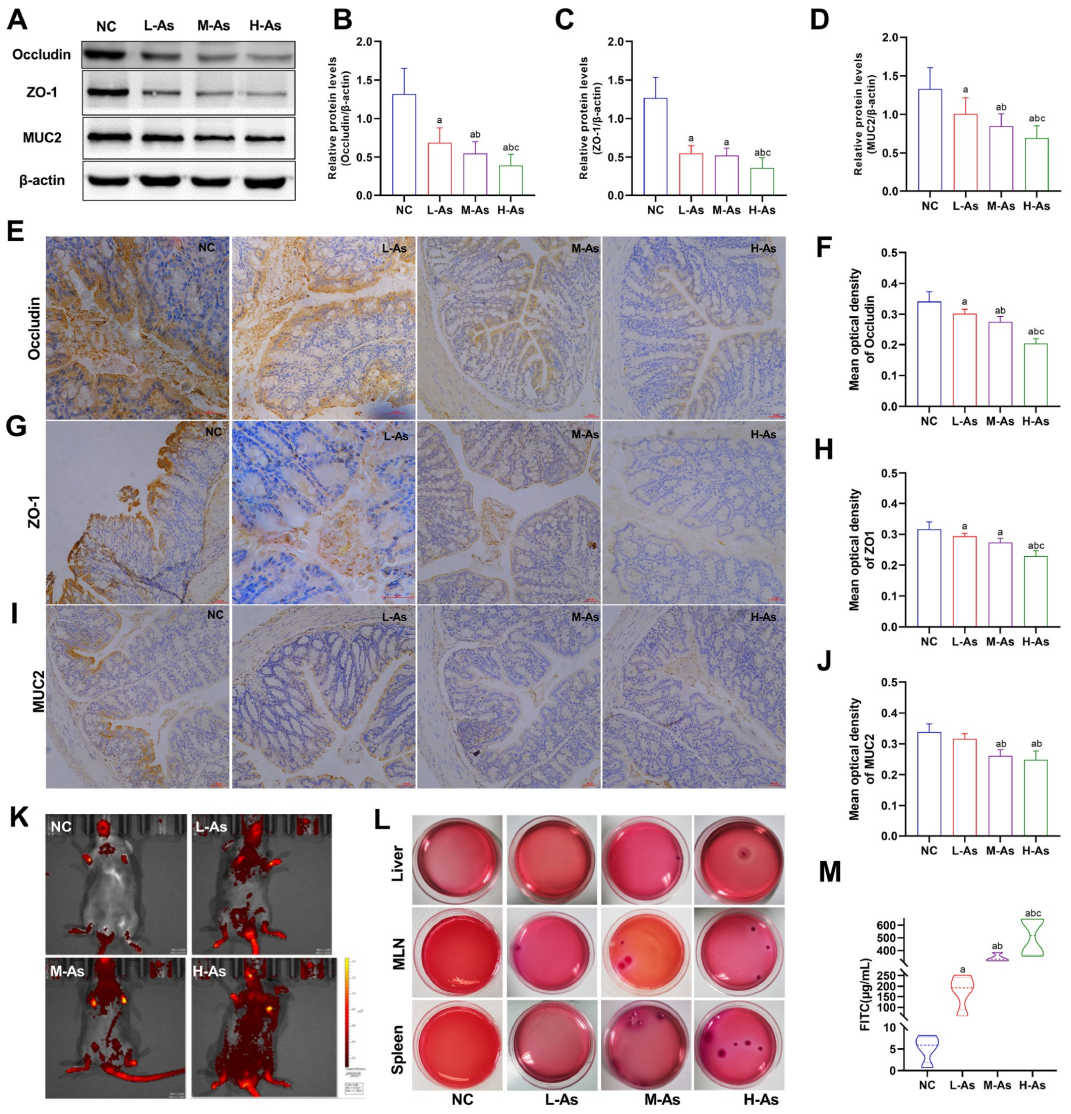
Figure 4 Effects of arsenic on intestinal barrier and intestinal permeability changes (A) Representative western blots showing the protein levels of Occludin, ZO-1, and MUC2 in colon tissues. (B–D) Quantitative and statistical analysis of Occludin, ZO-1, and MUC2 grey values from western blots, respectively. (E,G,I) Representative immunohistochemical images showing the protein levels of Occludin, ZO-1 and MUC2 in colon tissues (magnification 200×; scale bar: 50 μm). (F,H,J) Quantitative and statistical analysis of Occludin, ZO-1, and MUC2 levels determined by immunohistochemistry, respectively. (K) FITC distribution in the intestinal tract of mice by small animal imaging. (L) Bacterial quantities in the liver, MLNs, and spleen observed by MAC culture. (M) Content of FITC in the serum. Data are expressed as the mean±SD, n=4–6 for each group. aP<0.05 compared with the NC group; b P<0.05 compared with the L-As group; cP<0.05 compared with the M-As group.

To investigate the occurrence of intestinal barrier dysfunction after intestinal microbiota dysbiosis, we detected intestinal barrier markers, including Occludin, ZO-1, and MUC2. The results showed that, compared to those in NC mice, the protein expression levels of Occludin, ZO-1, and MUC2 were decreased in arsenic-exposed mice ( Figure 4A–D; *P*<0.05). Moreover, the protein levels of Occludin and MUC2 in the 4 mg/kg arsenic group were lower than those in the 1 mg/kg arsenic group and 2 mg/kg arsenic group ( *P*<0.05). Moreover, immunohistochemistry analysis revealed that arsenic decreased the Occludin ( Figure 4E,F), ZO-1 ( Figure 4G,H), and MUC2 ( Figure 4I,J) protein expressions in the colon tissues of mice.

Concurrently, as shown in Figure 4K,M, widely distributed FITC in the intestinal tract as well as in the serum was detected in arsenic exposed mice. In addition, arsenic administration increased the bacterial loads in the liver, MLNs, and spleen ( Figure 4L), indicating that arsenic increased intestinal epithelial permeability.

### Establishment of mouse model supplied with antibiotics

**Figure 5:**
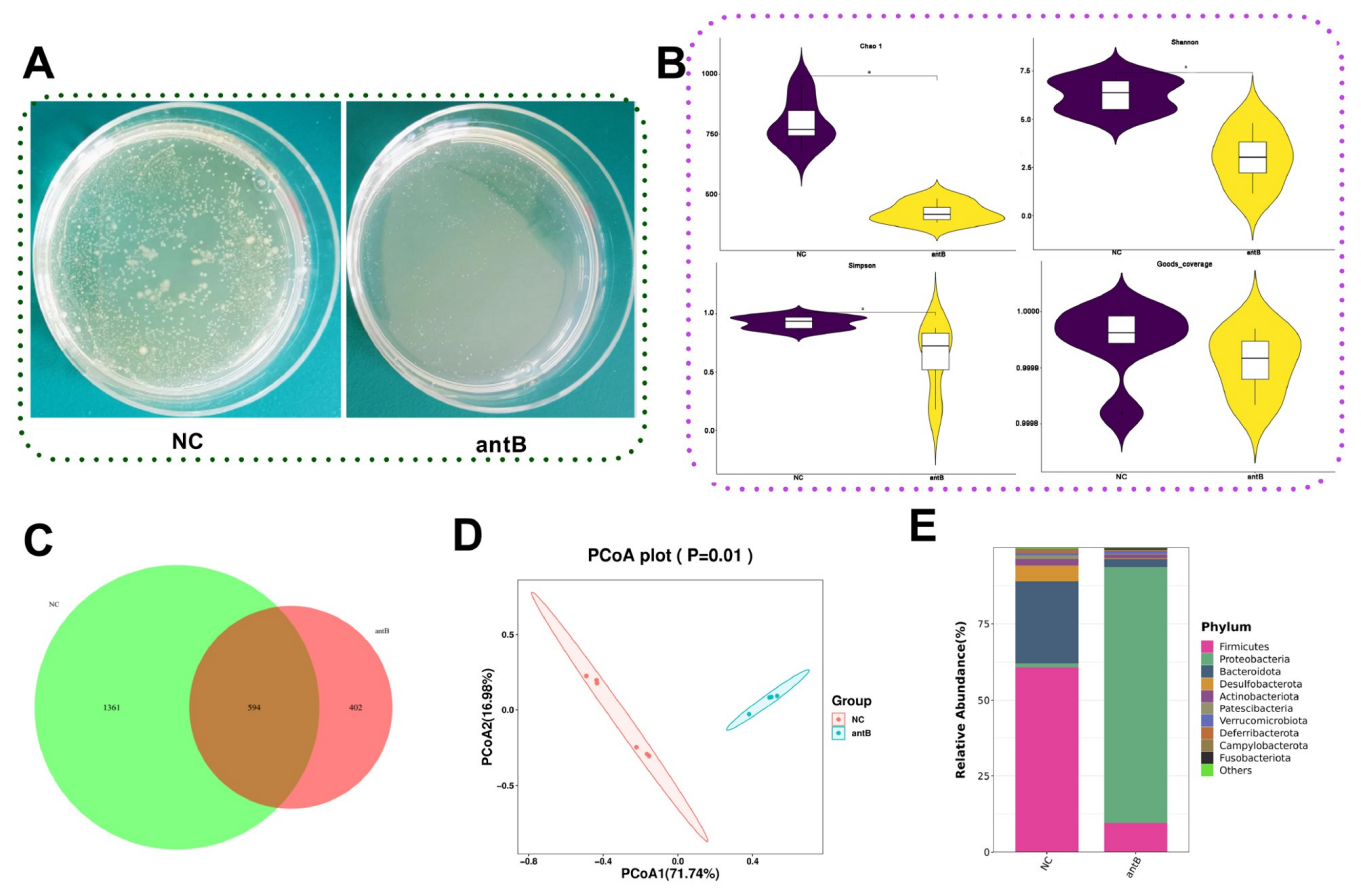
Figure 5 Appraisal of a pseudogerm-free mouse model (A) Bacterial loads in feces detected by BHIA culture. (B) Alpha diversity. (C) Venn diagram. (D) Principal coordinate analysis (PCoA) of weighted UniFrac distances. (E) Relative abundance of gut flora at the phylum level. NC: normal control; antB: treated with antibiotics. aP<0.05 compared with the NC group.

Fresh feces were collected from the control and antibiotic treatment (antB) groups of mice for microbe culturing and bacterial 16S rDNA sequencing. Compared to the NC group, the antB group exhibited a greater decrease in the total number of microbe colonies ( Figure 5A), Chao1 index, Shannon index, and Simpson index ( Figure 5B). However, no differences in Good’s coverage indices were found between the control animals and the antB group animals ( Figure 5B). The number of OTUs in the antB mice (402) was lower than that in the NC group (1361) ( Figure 5C). In terms of β-diversity, the composition of intestinal microbiota was different between the NC group and the antB group ( Figure 5D).

In addition, the relative abundances of intestinal flora differed between the NC and antB groups at the phylum level. The intestinal flora in NC mice included *Firmicutes* and *Bacteroidetes*, which substantially decreased after antibiotic supplementation. Remarkably, there was an abundance of *Proteobacteria* in the antB group ( Figure 5E). Overall, these data suggest that antibiotic supplementation can reduce the abundance of intestinal microbes in normal mice.

### The effect of fecal transplants on liver function in recipient mice

**Figure 6:**
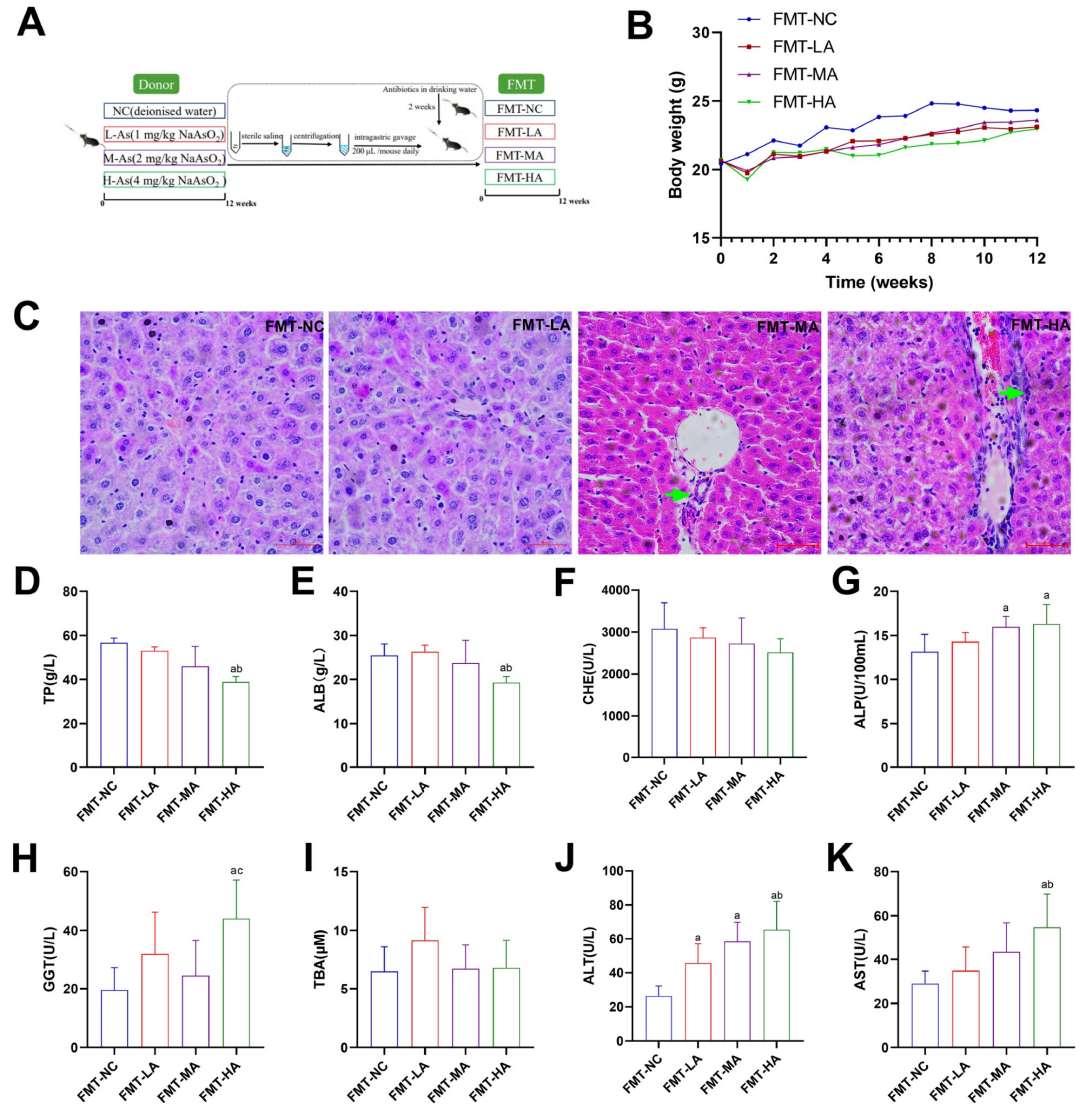
Figure 6 Hepatic histopathological and serum biochemical changes in mice gavaged with feces from arsenic-treated mice (A) The specific animal groups and administration methods. (B) Body weight changes. (C) Liver pathological changes (magnification 400×; scale bar: 50 μm). The green arrows represent inflammatory cell infiltration. (D) TP content. (E) ALB content. (F) CHE activity. (G) ALP activity. (H) GGT activity. (I) TBA content. (J) ALT activity. (K) AST activity. Data are expressed as the mean±SD; n=4–6 for each group. aP<0.05 compared with the FMT-NC group; bP<0.05 compared with the FMT-LA group.

To further confirm whether disturbances in the gut microbiome play a decisive role in the pathophysiology of liver under arsenic exposure, stool from mice exposed to arsenic was transferred to antibiotic-treated mice in the present study ( Figure 6A). Figure 6B showed that the differences in body weight among the FMT-NC, FMT-LA, FMT-MA, and FMT-HA mice were not statistically significant ( *P*>0.05). H&E staining results revealed inflammatory cells in the liver tissue of the recipient mice after FMT ( Figure 6C). Moreover, compared with those in the FMT-NC group, the ALP, GGT, ALT, and AST levels (Figure6 G,H,J,K, *P*<0.05) in the FMT-HA group were greater, whereas the serum levels of TP and ALB were lower ( Figure 6D,E), indicating that, FMT from arsenic-exposed mice damaged liver function in the recipient mice.

### Perturbation of the intestinal flora contributes to the pathogenesis of liver damage in arsenic-exposed mice

**Figure 7:**
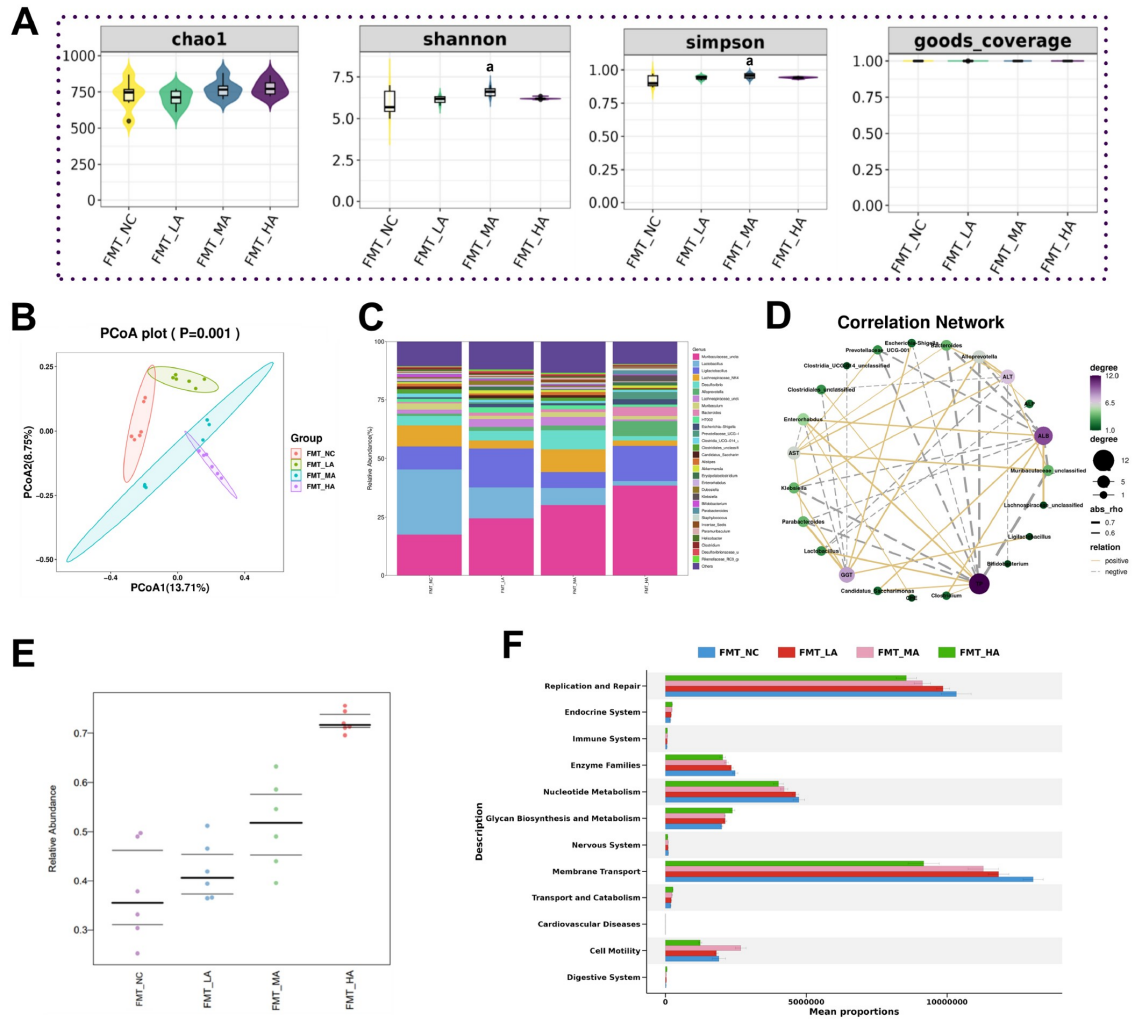
Figure 7 Gut microbiota dysbiosis in mice that received fecal transfer from arsenic-exposed mice (A) Accumulation curves and alpha diversity. (B) Principal coordinate analysis (PCoA) of weighted UniFrac distances. (C) Community structure analysis of each group at the genus level. (D) The correlation between liver function indicators and microbiota composition. (E) The potential pathogenicity of gut microbiota in pseudogerm-free mice that received fecal transfer from arsenic exposure mice. (F) Microbial gene functions predicted by PICRUSt. aP<0.05 compared with the FMT-NC group.

The intestinal microbiota of the recipient mice was determined by 16S rDNA sequencing. The Shannon index and Simpson index showed increased bacterial diversity in FMT-MA mice ( Figure 7A). As expected, PCoA at the genus level clustered the microbiota compositions among the mice in the FMT-NC group and the FMT-LA, FMT-MA, and FMT-HA groups, and there were significant differences among these groups ( Figure 7B). Moreover, at the genus level, *Muribaculaceae_unclassified*, *Ligilactobacillus*, *Scherichia-Shigella*, *Klebsiella*, *Bacteroides*, and *Alloprevotella* were confirmed to be abundant, while *Lactobacillus*, *Lachnospiraceae_NK4A136_ group*, and *Desulfovibrio* were shown to be more deficient in FMT-HA mice ( Figure 7C).

Figure 7D shows the correlation analysis between FMT fecal bacteria and liver function parameters. The levels of FMT arsenic-enriched gut bacteria, such as *Muribaculaceae_unclassified* , *Ligilactobacillus*, *Escherichia-Shigella*, *Klebsiella*, *Bacteroides*, and *Alloprevotella* were positively correlated with the serum levels of GGT, ALT, and AST, but negatively correlated with TP and ALB ( *P*<0.05). Additionally, the abundances of arsenic-reducing gut bacteria, such as *Lactobacillus and Lachnospiraceae_unclassified*, were positively correlated with the serum parameters TP and ALB, but negatively correlated with ALP and ALT ( *P*<0.05).

The gut microbiota of mice that received stool translocation from arsenic-exposed mice had greater potential pathogenicity ( Figure 7E). Moreover, gut microbial gene functions in mice that received fecal transfer included the digestive system, cardiovascular diseases, cell growth and death, glycan biosynthesis and metabolism, and nervous system ( Figure 7F). These data indicated that arsenic-induced hepatotoxicity might be transferrable by fecal transplant.

### Fecal transplantation induces gut damage in recipient mice

**Figure 8:**
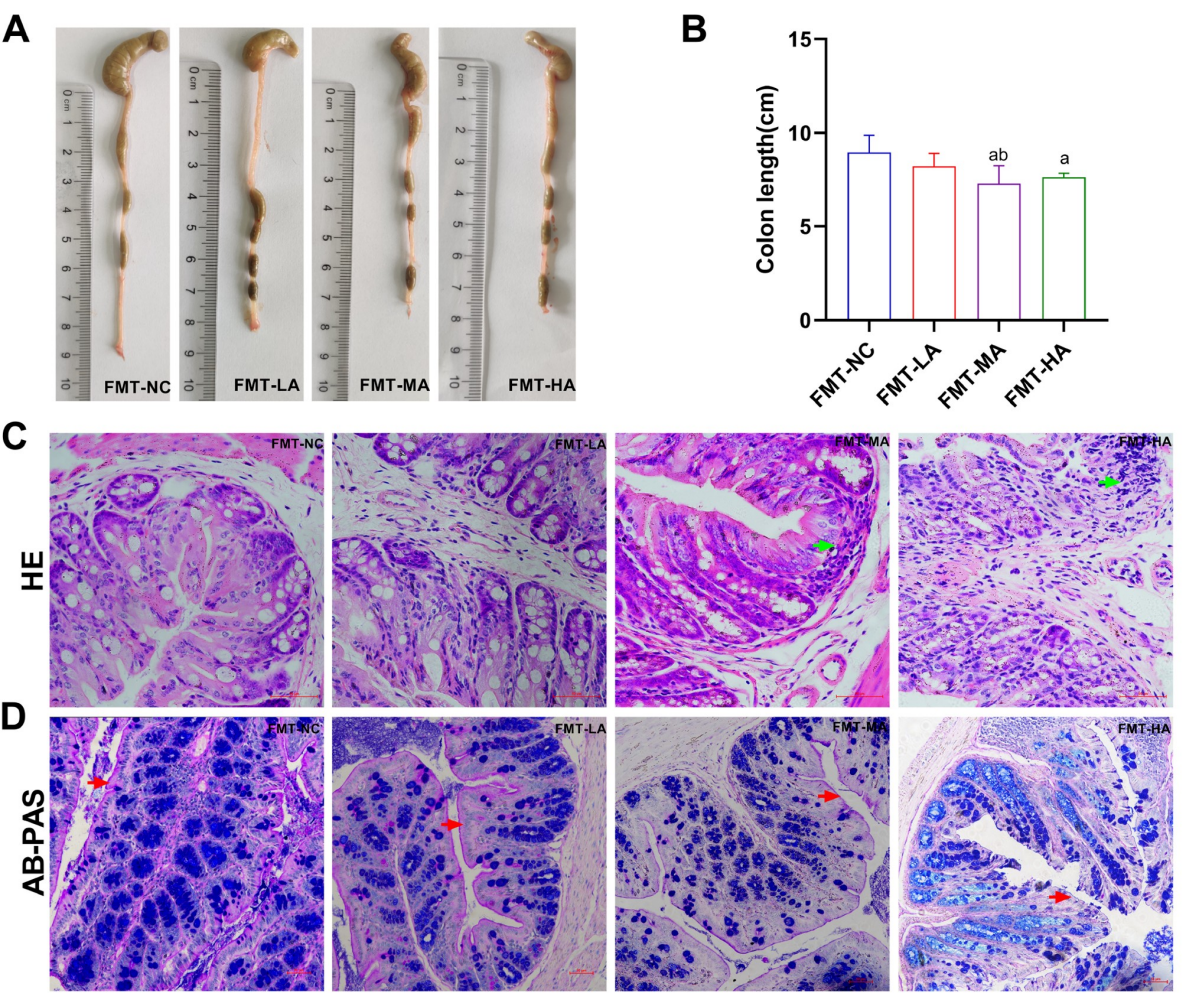
Figure 8 Gut histopathological changes in mice gavaged with feces from arsenic-treated mice (A) Colon length. (B) Colon length (bar chart). (C) Pathological changes in the colon (magnification 400×; scale bar: 50 μm). The green arrows indicate inflammatory cell infiltration. (D) AB-PAS staining of the colonic mucosa (magnification 200×; scale bar: 50 μm), the red arrows indicate mucin layer. Data are expressed as the mean±SD; n=4–6 for each group. a P<0.05 compared with the FMT-NC group; bP<0.05 compared with the FMT-LA group; cP<0.05 compared with the FMT-MA group.

To investigate whether the gut flora contribute to the pathogenesis of gut damage, fecal microbiota transfer experiments were further performed. Compared to the FMT-NC mice, the mice that underwent arsenic-manipulated gut microbiota transplantation had shorter colon lengths ( Figure 8A,B), greater inflammatory cell infiltration ( Figure 8C), and thinner mucin layers ( Figure 8D). These results suggested that arsenic-induced liver damage is related to the regulation of the intestinal flora.

### FMT induces intestinal barrier dysfunction and increases intestinal permeability in recipient mice

**Figure 9:**
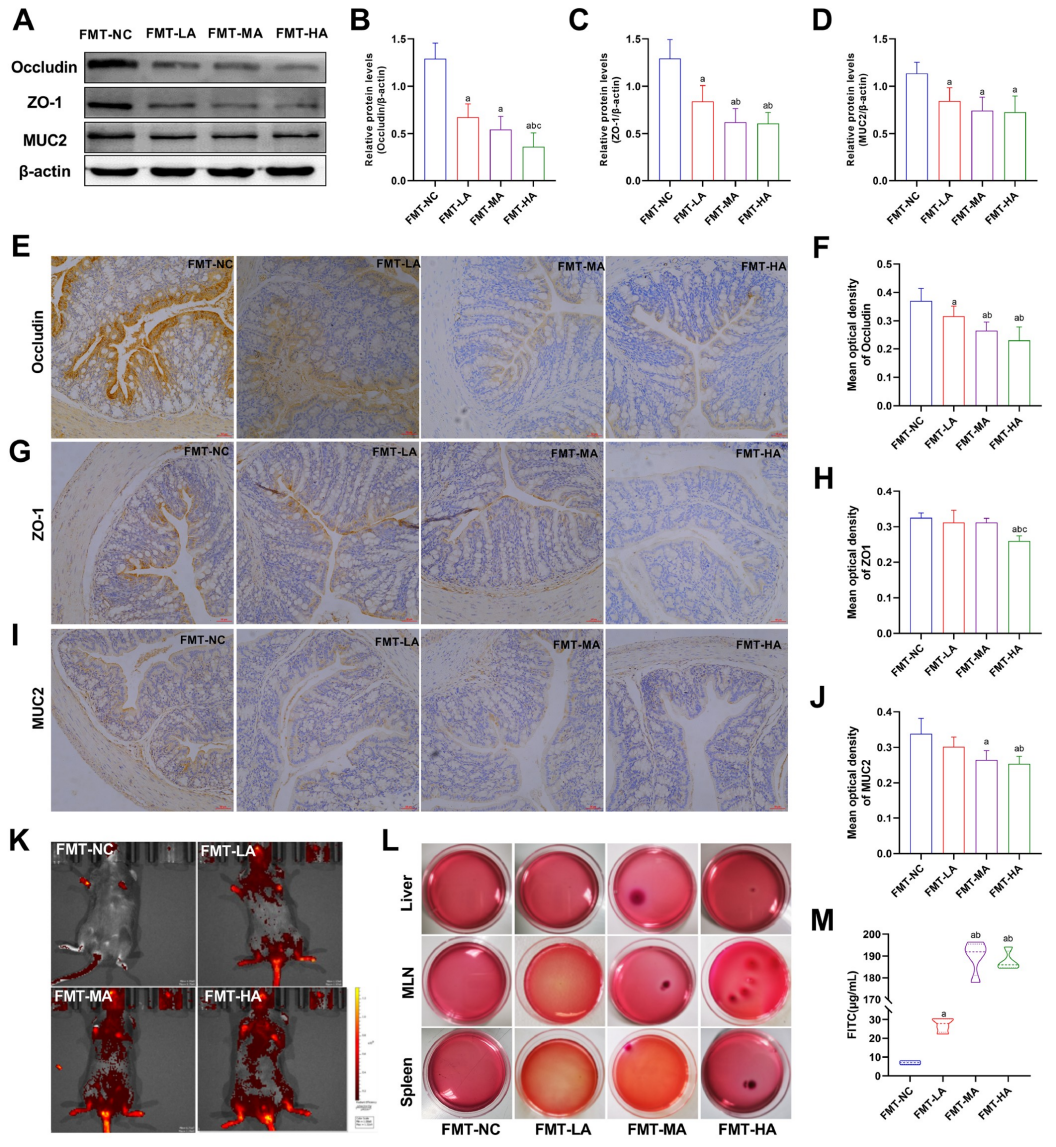
Figure 9 Intestinal barrier and intestinal permeability in mice gavaged with feces from arsenic-treated mice (A) Representative western blots showing the protein levels of Occludin, ZO-1, and MUC2 in colon tissues. (B-D) Quantitative and statistical analysis of Occludin, ZO-1, and MUC2 grey values from western blots, respectively. (E,G,I) Representative immunohistochemical images showing the protein levels of Occludin, ZO-1, and MUC2 in colon tissues (magnification 200×; scale bar: 50 μm). (F,H,J) Quantitative and statistical analysis of Occludin, ZO-1, and MUC2 levels determined by immunohistochemistry, respectively. (K) FITC distribution in the intestinal tract of mice by small animal imaging. (L) Bacterial loads in liver, MLNs and spleen detected by MAC culture. (M) Content of FITC-dextran in the serum. Data are expressed as the mean±SD, n=4-6 for each group. aP<0.05 compared with the NC group; b P<0.05 compared with the L-As group; cP<0.05 compared with the M-As group.

To investigate the occurrence of intestinal barrier dysfunction and intestinal permeability after fecal microbiota transfer from arsenic-exposed mice, we detected intestinal barrier markers in colon and FITC levels in the serum and intestinal tract. Compared with those in the FMT-NC group, the expression levels of the tight junction proteins Occludin, ZO-1, and MUC2 in FMT-MA and FMT-HA mice were lower after the transfer of feces from mice treated with arsenic ( Figure 9A‒J). These findings demonstrated that the transfer of stool from arsenic-exposed mice promoted the loss of colonic barrier integrity. Compared with the FMT-NC group, mice that received arsenic-manipulated gut microbiota transplantation exhibited increased FITC levels in the serum and intestinal tract ( Figure 9K,M) and increased bacterial loads in the liver, MLNs, and spleen ( Figure 9L). These data indicated that the transfer of feces from arsenic-exposed mice promoted intestinal epithelial permeability.

## Discussion

Arsenic is an environmental pollutant that can cause severe and extensive multiorgan system toxicity, which has attracted widespread attention. Liver is a key organ affected by arsenic toxicity. Epidemiological studies have shown that coal-burning and drinking water arsenic exposure increase the risk of liver damage [ 3, 31]. In mice, exposure to arsenic for 8 weeks induced liver injury [32]. However, the mechanisms involved in the induction of hepatotoxicity by arsenic are unclear. In the present work, following 12 weeks of arsenic treatment, serum levels of ALP, GGT, TBA, AST, and ALT in the arsenic-exposed mice were greater than those in the control animals. When liver injury occurs, the serum liver parameter levels increase [33]. Our findings indicated that arsenic induced liver injury in mice, which was further confirmed by liver histopathology.

The origin of liver damage caused by exposure to arsenic remains unclear, and it should be noted that the gut flora has been regarded as one of the main causes of liver damage. The gut microbiota plays a vital role in gut and the extra-gut organs [34]. A previous study suggested that arsenic-induced hepatotoxicity is a multistep process associated with the gut-liver axis [15]. It has been found that arsenic alters the intestinal microbiota, fecal concentration of short-chain fatty acids, and plasma metabolites in mice [ 35, 36]. Arsenic exposure is associated with the occurrence and development of gastrointestinal symptoms such as intestinal injury [37]. In addition, arsenic obviously impairs intestinal structure, morphology, and function in mice [ 38, 39], resulting in dysbiosis of the intestinal microbiota. In the present study, HE and AB-PAS staining revealed inflammatory cell infiltration and a thinner mucin layer in arsenic-exposed animals.

Previous studies on arsenic exposure have shown some changes in the gut microbiota [ 28, 40– 42]. In this study, we found that arsenic induced an increase in the abundance of *Escherichia-Shigella*, *Bacteroides*, *Blautia*, and *Lachnospiraceae_unclassfied* in mice. However, compared to those in NC animals, the relative abundances of *Muribaculum*, *Lactobacillus*, and *Muribaculaceae_unclassfied* decreased in the animals exposed to arsenic. Then, we profiled arsenic-related microbial gene features, including membrane transport, amino acid metabolism, energy metabolism, lipid metabolism, and cellular processes and signaling. Our data emphasize the significance of probing arsenic-induced hepatotoxicity-related gut microbial changes at the functional level.

The complete intestinal barrier plays a vital role in immunity, metabolism, and digestive tract absorption [ 43, 44]. Consistent with previous studies [ 39, 45], in comparison to the control mice, the arsenic exposure groups showed significantly decreased levels of Occludin and ZO1, which are important proteins closely associated with the intestinal barrier [46]. In addition, it should be noted that the intestinal mucosa of mice treated with arsenic results in marked down-regulation of MUC2, which is a vital element of the intestinal immune barrier [ 43, 47]. Increased intestinal permeability was observed in subchronic arsenic-exposed mice in the present study, resulting in dysbiosis of the intestinal barrier. Loss of the intestinal barrier leads to the translocation of harmful microbes to the liver and blood, resulting in liver damage [48]. In the present study, we observed that the distribution of FITC in the arsenic-exposed animals was greater not only in the intestinal tract but also in the serum. In addition, arsenic exposure increased the bacterial contents in the liver, MLNs, and spleen, indicating that arsenic increased intestinal epithelial permeability. The intestinal microbiota is one of the main components of the intestinal mucosal barrier. The disturbance of the intestinal flora is a critical factor that results in the disruption of the intestinal barrier [49]. Therefore, we speculated that the disturbance of the intestinal microbiota by arsenic may be one of the causes of intestinal barrier disruption, which might cause harmful gut microbiota to intrude into intestinal lamina propria, translocate into the blood, activate inflammatory reactions, and promote liver damage.

Currently, FMT has been applied to discriminate noncausal or causal relationships between the intestinal microbiota and diseases [50]. On the one hand, previous studies confirmed the beneficial effects of FMT in the treatment of diseases, such as *Clostridium difficile* infection [51] and liver disease [52]. On the other hand, FMT has been applied to verify the effects of the intestinal flora on many conditions, such as depressive disorder [22], hypertension [25], diabetes [26], and liver damage [ 53, 54]. Therefore, to evaluate the causal relationship between the intestinal flora and liver damage in arsenic-exposed mice, the intestinal microbiota, which originates from the intestinal microbiota of arsenic-exposed mice, was transferred to mice with their gut microbiota removed. Currently, an antibiotic treatment mice model, which is constructed by supplementing the intestinal tract of subject animals with mixed antibiotics to clean the gut flora by a large majority, has been extensively applied to explore the gut microbiota [55]. In the present work, we successfully established a pseudogerm-free mouse model, so it is reasonable to evaluate the causal relationship between the intestinal microbiota and disease in this model. Under the same experimental conditions, compared with control (FMT-NC) transplantation, arsenic-manipulated gut microbiota transplantation markedly increased the levels of ALP, GGT, AST, and ALT but decreased the levels of TP and ALB. Obvious differences were observed in the liver histopathology of the mice that were gavaged with feces from arsenic-treated and normal mice, which indicated that FMT from the arsenic groups significantly deteriorated liver function in recipient mice.

We further found that the composition of the intestinal microbiota clearly differed among the four groups based on PCoA. Compared with those in the control group, the relative abundances of *Ligilactobacillus*, *Escherichia-Shigella*, and *Klebsiella* in the FMT-HA group increased. However, the levels of many beneficial intestinal bacteria, such as *Lactobacillus* and *Lachnospiraceae_NK4A136_group*, were decreased in the FMT-HA group compared with those in the FMT-NC group.

Moreover, the gut microbiota in mice after receiving stool transfer from arsenic-exposed mice had greater potential pathogenicity. In the present study, FMT data indicated that the abundances of *Escherichia-Shigella* and *Klebsiella* were increased. These findings are consistent with the sequencing results of the intestinal microbiota in arsenic-exposed mice. FMT treatment increased many potentially harmful bacterial species, such as *Klebsiella* and *Escherichia_Shigella*, which are positively correlated with inflammation in the liver [53]. In addition, it has been pointed out that the proinflammatory harmful bacteria *Escherichia_Shigella* is related to metabolic dysbiosis in individuals with obesity [56]. Furthermore, our present results demonstrate that FMT from the arsenic-exposed groups decreases the abundance of *Lactobacillus* in pseudogerm-free mice, which suggests that the adverse effects of FMT may be attributed to decreased levels of beneficial bacteria and increased abundances of those harmful species. Previous studies proposed that *Lactobacillus* supplementation improves non-alcoholic fatty liver disease (NAFLD) by regulating the intestinal flora and inflammation [ 57, 58]. Other studies have also demonstrated that *Lactobacillus* is effective in improving liver function [59] .

Then, gut histopathology and the intestinal mucosal barrier were detected to evaluate intestinal epithelial permeability. Interestingly, FMT affected the epithelial structure and significantly decreased the levels of the intestinal physical and immune barrier proteins Occludin, ZO1 and MUC2 in the FMT-MA and FMT-HA mouse colon. Moreover, intestinal barrier dysfunction and an increase in gut epithelial permeability were found in the mice that received fecal transfer from the arsenic exposure groups.

Nevertheless, this study has some limitations. Due to the effect of the intestinal flora on arsenic metabolism as well as the different modes and types of arsenic metabolism *in vivo*, there are significant individual differences in susceptibility to arsenic-induced injuries [60], and these factors can have various effects on the toxicological effects of arsenic. Therefore, based on the current study, the definite effect (causal from noncausal) of FMT could not be confirmed, and more systematic and specific validation studies are needed in follow-up studies.

In conclusions, based on the data obtained in the present study, we can conclude that subchronic exposure to arsenic can affect the homeostasis of intestinal microbiota and subsequently induce intestinal barrier dysfunction, triggering an increase in intestinal permeability and causing damage to liver tissues in mice. More importantly, gut flora dysbiosis can be transferred to pseudogerm-free mice via FMT and trigger liver damage in the recipient mice. This study demonstrates that changes in the intestinal microbiota of mice exposed to arsenic contribute to liver damage.
